# Do Abstinence-Plus Interventions Reduce Sexual Risk Behavior among Youth?

**DOI:** 10.1371/journal.pmed.0040276

**Published:** 2007-09-18

**Authors:** Shari L Dworkin, John Santelli

## Abstract

The authors discuss the policy questions arising from a new study on "abstinence-plus" interventions for reducing HIV risk behavior among youth in high-income countries.

Abstinence until marriage has emerged as a primary policy goal in efforts to promote adolescent sexual and reproductive health—in the United States and increasingly world-wide. While few would argue with abstinence as a personal choice (no one should be forced to have sex), there are serious questions about whether government promotion of abstinence should be a public health goal. Marriage is not free of HIV risk. Early marriage—particularly of young women to older, sexually experienced men—carries a substantial risk of HIV infection. Around the globe, a young woman's primary risk of HIV infection is often through sex with her husband [1].

Sexual intercourse is almost universally initiated during adolescence worldwide. By age 20, 77% of young people in the US have initiated sex, and 76% have had premarital sex [2]. By age 25, over 90% people have had sex, with only about 3% waiting for marriage. Over the past 40 years, the median age at first intercourse has dropped (and stabilized) to age 17 in most developed countries [3]. Even more dramatic, however, has been the increasing age at marriage. In the United States, between 1970 and 2002 the median age at first sex for young women fell from 19 to 17 years, while the median age at marriage rose from 20 to 25 [4]. These enormous demographic changes stand in sharp contrast to the modest impact of health education in promoting abstinence or the small decline during the 1990s in sexual activity.

## Objections to Abstinence-Only Education

The most vociferous criticism of abstinence as a public health goal has been directed toward abstinence-only education (i.e., education that presents abstinence as the best and only solution and restricts information about other prevention strategies such as condom use). Scientific and ethical objections to such education have come from the major professional organizations in the US focused on adolescent health, including the American Academy of Pediatrics, the Society for Adolescent Medicine, the American Medical Association, and the American Public Health Association [5–8].

**Linked Research Article**

This Perspective discusses the following new study published in *PLoS Medicine:*

Underhill K, Operario D, Montgomery P (2007) Systematic review of abstinence-plus HIV prevention programs in high-income countries. PLoS Med 4(9): e275. doi:10.1371/journal.pmed.0040275

In their systematic review, Underhill and colleagues found that abstinence-plus programs appear to reduce short-term and long-term HIV risk behavior among youth in high-income countries.

Two recent systematic reviews of abstinence-only curricula suggest that the best implemented and evaluated programs fail to delay initiation of sexual intercourse or to produce other demonstrable reductions in HIV risk behaviors [9,10]. A six-year longitudinal study of virginity pledgers found short-term delays in sexual intercourse but no impact on laboratory-verified sexually transmitted infection [11]. A 2004 US Congressional review found that 11 of the 13 most frequently used abstinence-only curricula contained false, misleading, or distorted information—including inaccurate information about contraceptive effectiveness, risks of abortion, and other scientific errors [12]. Another review found that curricula often provide misinformation about condoms and contraception [13].

Moreover, abstinence-only promotion has undermined comprehensive sexuality education (i.e., complete, age-appropriate education on human sexuality including abstinence and risk reduction) within US public schools, harmed other critical public health efforts such as family planning programs, and created disarray in US efforts to prevent HIV globally [4,14–17]. For example, during the period of increasing US emphasis on abstinence, sharp declines have occurred in the percentage of teachers in US public schools who teach about birth control and the number of students who report receiving such education [14,15].

Finally, promotion of abstinence as a sole option for adolescents and young adults raises serious human rights concerns, because it involves withholding health- and life-saving information from teenagers. Access to complete and accurate HIV/AIDS and sexual health information is recognized as a basic human right by many international agreements [4,17]. Governments have an obligation to ensure complete and accurate information in publicly supported programs, and adolescents have a right to expect health education provided in public schools to be scientifically accurate and complete.

## A New Study of “Abstinence-Plus” Interventions

A new systematic review by Kristen Underhill and colleagues in this issue of *PLoS Medicine* addresses a related approach to abstinence promotion—abstinence-plus interventions [18]. In abstinence-plus education, participants are given a hierarchy of safe-sex strategies. At the top of the hierarchy is the promotion of sexual abstinence as the safest route to HIV prevention. Recognizing that some participants will not be abstinent, abstinence-plus approaches encourage individuals to also use condoms and to adopt other safer-sex strategies.

Abstinence-plus approaches should be distinguished from other comprehensive approaches. Comprehensive approaches may not place abstinence as an approach “above” other strategies. Those who favor comprehensive approaches may, in fact, oppose abstinence-plus interventions for undermining condom use and other safer-sex strategies. On the other hand, those who favor abstinence-only strategies may criticize abstinence-plus approaches as potentially undermining the promotion of abstinence. Underhill and her coauthors therefore place themselves squarely into a contentious intellectual space in setting out to evaluate the impact of abstinence-plus programs on youth behaviors in high-income countries. In their systematic review, the authors included randomized and quasi-randomized controlled trials of abstinence-plus interventions, including trials among participants of negative or unknown HIV serostatus. They found that 23 of 39 abstinence-plus trials reported a protective effect on at least one sexual behavior, including abstinence, condom use, and unprotected sex. No trials found adverse impacts of abstinence-plus interventions on any of these behavioral outcomes. Program settings and formats varied and mainly included schools and community facilities. Programs were mainly delivered in small or large group formats.

At first glance, the finding that 23 of 39 trials reported a protective effect on sexual risk behaviors appears promising for HIV/AIDS prevention efforts. Twelve trials included in the review assessed participants' self-reported frequency of unprotected vaginal sex, and six of these twelve trials found that abstinence-plus interventions had a protective effect. Twenty-one trials evaluated the incidence of any protected or unprotected vaginal sex, and five of these 21 trials showed a significant protective effect of the interventions. Thirteen trials assessed the frequency of partners, and of these, four found that abstinence-plus interventions reduced the number of partners. Fourteen of the 26 trials that measured condom use found that abstinence-plus interventions were associated with increased condom use, while four of the 19 trials that assessed sexual initiation found a protective effect for these interventions (but mainly among females). Overall, abstinence-plus programs did not increase HIV risks among youth in any study.

The authors contrast the impact of abstinence-plus programs to the lack of positive impact of abstinence-only programs in their own recent systematic review [9]. Given the exclusion of abstinence-plus programs from federal abstinence funding, the authors rightfully declare that it may be “prudent to reconsider these resource allocation policies” [18].

While the overall trends in the new study in *PLoS Medicine* are clear, making sense of the detailed results and interpreting this morass of information is no easy task. Few studies included in the systematic review adequately defined abstinence, trials rarely used widely acceptable intention-to-treat analyses, attrition was quite high in several trials, few trials had clear descriptions of what “usual care” meant in control arms, and no studies directly compared abstinence-only to abstinence-plus trials. The authors clearly note these definitional and methodological weaknesses.

## Public Health, Ethical, and Clinical Implications

The authors argue that in their study of abstinence-plus interventions, “the promotion of abstinence did not appear to detract from the programs' condom promotion message” [18]. However, many programmatic and policy questions remain.

A list of programmatic questions is shown in Box 1. Kirby and colleagues have identified from effective comprehensive programs 17 characteristics that can guide the development of new programs and the implementation and adaptation of existing effective programs [10]. From a policy viewpoint, we must examine the extent to which abstinence-plus programs have a public health emphasis or are taking a religious approach to a perceived moral problem. Moreover, if abstinence-plus programs are effective in both high- and low-income countries, why does the US restrict public funding for such programs? A final critical policy question concerns the length of behavioral impact. If the primary benefit of abstinence-plus programs is a modest delay in initiation of sexual intercourse (e.g., three to six months), their potential long-term value is extremely limited.

**Box 1. Programmatic Questions About Abstinence-Plus Interventions**

- What are the characteristics of effective abstinence-plus programs?
- Are there significant mediational variables (e.g., those variables that are responsible for the ultimate behavioral outcome) to focus on in abstinence-plus programs?
- Can multiple outcomes be impacted simultaneously?
- What is the relative emphasis on abstinence versus other prevention strategies in abstinence-plus programs?
- Do abstinence-plus programs provide balanced and scientifically accurate information to teens?
- Do these programs undermine confidence in condoms and contraception?
- Do programs conflate gender stereotypes with scientific fact (e.g., in reviews of abstinence-only programs, content was said to undermine girls' achievements, make statements that girls are naturally weaker, or put forward assumptions that men are sexually aggressive by nature)? [12]
- Do programs reinforce race and class stereotypes (e.g., the inclusion of abstinence-until-marriage as the highest “standard of human conduct” emerged in welfare-to-work policies)?
- Do programs provide unrealistic information about the success of abstinence or the risk of HIV and other sexually transmitted infections within marriage?
- How are gay, lesbian, bisexual, transgendered, and questioning populations affected by the emphasis on abstinence or abstinence until marriage?

The US influences policy and program choices in many other countries. In developing countries, which bear the greatest burden of HIV, the US government abstinence policy has been shown to reshape national level HIV/AIDS prevention programs by restricting information about condoms and other risk reduction strategies [16,17]. As a result, national programs are developed with separate components and different goals—some promoting condoms and risk reduction, some prioritizing abstinence but providing information about risk reduction, and others promoting abstinence and denigrating condoms. What will be the result?

## References

[pmed-0040276-b001] Hirsch JS, Meneses S, Thompson B, Negroni M, Pelcastre B (2007). The inevitability of infidelity: Sexual reputation, social geographies, and marital HIV risk in rural Mexico. Am J Public Health.

[pmed-0040276-b002] Finer LB (2007). Trends in premarital sex in the United States, 1954–2003. Public Health Rep.

[pmed-0040276-b003] Teitler JO (2002). Trends in youth sexual initiation and fertility in developed countries: 1960–1995. Ann Am Acad Pol Soc Sci.

[pmed-0040276-b004] Santelli J, Ott MA, Lyon M, Rogers J, Summers D (2006). Abstinence and abstinence-only education: A review of U.S. policies and programs. J Adolesc Health.

[pmed-0040276-b005] American Academy of Pediatrics Committee on Psychosocial Aspects of Child and Family Health and Committee on Adolescence (2001). Policy statement: Sexuality education for children and adolescents. Pediatrics.

[pmed-0040276-b006] American Medical Association (2007). H-170.968 Sexuality education, abstinence, and distribution of condoms in schools. http://www.ama-assn.org/apps/pf_new/pf_online?f_n=browse&doc=policyfiles/HnE/H-170.968.HTM.

[pmed-0040276-b007] American Public Health Association (2006). Abstinence and U.S. abstinence-only education policies: Ethical and human rights concerns policy statement. Policy number 200610. http://www.apha.org/advocacy/policy/policysearch/.

[pmed-0040276-b008] Santelli J, Ott MA, Lyon M, Rogers J, Summers D (2006). Abstinence-only education policies and programs: A position paper of the Society for Adolescent Medicine. J Adolesc Health.

[pmed-0040276-b009] Underhill K, Montgomery P, Operario D (2007). Sexual abstinence only programmes to prevent HIV infection in high-income countries: Systematic review. BMJ.

[pmed-0040276-b010] Kirby D, Laris BA, Rolleri LA (2007). Sex and HIV education programs: Their impact on sexual behaviors of young people throughout the world. J Adolesc Health.

[pmed-0040276-b011] Bruckner H, Bearman PS (2005). After the promise: The STD consequences of adolescent virginity pledges. J Adolesc Health.

[pmed-0040276-b012] United States House of Representatives Committee on Government Reform—Minority Staff Special Investigations Division (2004). The content of federally funded abstinence-only education programs. http://oversight.house.gov/documents/20041201102153-50247.pdf.

[pmed-0040276-b013] Santelli J (2007). Declaration of John S. Santelli, MD, MPH. http://www.aclu.org/reproductiverights/sexed/29486lgl20070426.html.

[pmed-0040276-b014] Duberstein-Lindberg L, Santelli JS, Finer LB, Singh S (2006). Changes in formal sex education: 1995 and 2002. Perspect Sex Reprod Health.

[pmed-0040276-b015] Darroch JE, Landry DL, Singh S (2000). Changing emphases in sexuality education in U.S. public secondary schools. Fam Plann Perspect.

[pmed-0040276-b016] U.S. Government Accountability Office (2006). Global health: Spending requirement presents challenges for allocating prevention funding under the President's emergency plan for AIDS relief. http://www.gao.gov/new.items/d06395.pdf.

[pmed-0040276-b017] Cohen J, Tate T (2005). The less they know, the better: Abstinence-only HIV/AIDS programs in Uganda. Human Rights Watch. http://hrw.org/reports/2005/uganda0305/uganda0305.pdf.

[pmed-0040276-b018] Underhill K, Operario D, Montgomery P (2007). Systematic review of abstinence-plus HIV prevention programs in high-income countries. PLoS Med.

